# Comorbid Analysis of Genes Associated with Autism Spectrum Disorders Reveals Differential Evolutionary Constraints

**DOI:** 10.1371/journal.pone.0157937

**Published:** 2016-07-14

**Authors:** Maude M. David, David Enard, Alp Ozturk, Jena Daniels, Jae-Yoon Jung, Leticia Diaz-Beltran, Dennis. P. Wall

**Affiliations:** 1 Department of Pediatrics, Division of Systems Medicine, Stanford University, Stanford, California, United States of America; 2 Department of Biology, Stanford University, Stanford, California, United States of America; Swiss Federal Institute of Technology (ETH Zurich), SWITZERLAND

## Abstract

The burden of comorbidity in Autism Spectrum Disorder (ASD) is substantial. The symptoms of autism overlap with many other human conditions, reflecting common molecular pathologies suggesting that cross-disorder analysis will help prioritize autism gene candidates. Genes in the intersection between autism and related conditions may represent nonspecific indicators of dysregulation while genes unique to autism may play a more causal role. Thorough literature review allowed us to extract 125 ICD-9 codes comorbid to ASD that we mapped to 30 specific human disorders. In the present work, we performed an automated extraction of genes associated with ASD and its comorbid disorders, and found 1031 genes involved in ASD, among which 262 are involved in ASD only, with the remaining 779 involved in ASD and at least one comorbid disorder. A pathway analysis revealed 13 pathways not involved in any other comorbid disorders and therefore unique to ASD, all associated with basal cellular functions. These pathways differ from the pathways associated with both ASD and its comorbid conditions, with the latter being more specific to neural function. To determine whether the sequence of these genes have been subjected to differential evolutionary constraints, we studied long term constraints by looking into Genomic Evolutionary Rate Profiling, and showed that genes involved in several comorbid disorders seem to have undergone more purifying selection than the genes involved in ASD only. This result was corroborated by a higher dN/dS ratio for genes unique to ASD as compare to those that are shared between ASD and its comorbid disorders. Short-term evolutionary constraints showed the same trend as the pN/pS ratio indicates that genes unique to ASD were under significantly less evolutionary constraint than the genes associated with all other disorders.

## Introduction

Autism Spectrum Disorder (ASD) is a heritable developmental disorder that affects one in sixty-eight children [1]. Its prevalence is rising at an alarming rate, up from one in eighty-eight children in 2008 [2]. The scientific community has studied this disorder and shown that the burden of comorbidity is substantial. For example, analysis of health records indicates that over 10% of individuals diagnosed with ASD have bowel disorders, over 10% have epilepsy, over 5% present cranial anomalies, and over 2% schizophrenia [3].

There has already been a massive sequencing effort which has led to the discovery of many genetic biomarkers of Autism Spectrum Disorder [4–6], as well as its comorbid disorders. Genomic data on nearly 21,000 individuals with autism (12,694 SNP and array, 6,122 exomes and 2400 whole genomes) have been banked in the pursuit of genetic markers. These efforts are collectively beginning to sharpen the molecular picture of autism, which now includes at least sixty genes with variants of high interest [1,4]. Importantly, however, the symptoms of autism overlap with many other human diseases and conditions both neurological in nature and not. Whether this substantial overlap reflects convergence or common molecular pathologies, it strongly suggests that cross-disorder analysis will help prioritize known and identify new autism candidates. For example, known genes in the intersection between autism and many related conditions (behaviorally related, comorbid or both) may represent nonspecific indicators of dysregulation and impairment that do no relate to the root causes of Autism. Conversely, there may exist genes unique to autism that have not been commonly associated with other diseases and may play a more causal role.

One way to perform a cross-disorder analysis of the results is through systematic screening of all genes already associated with ASD and its comorbid disorders. Identifying genes uniquely related to ASD will allow us to better analyze gene functions and identify complex disease genes by describing metabolic and regulatory pathways unique to this condition.

But beyond the description of functionally distinct pathways underlying autism, can we find an evolutionary signature intrinsic to the sequences unique to ASD in comparison to those of comorbid diseases? By comparing the genes involved in ASD and its closely related disorders, we can determine whether the sequence of these characterized genes have been subjected to differential evolution constraints, either purifying or positive selection during the evolutionary timeframe for mammals or in the more recent evolution of humans. Such analysis would not only define evolutionary trends of ASD genes, but also allow us to relate this to the molecular pathology of autism.

In the present work, we performed an automated extraction of genes associated with ASD and its comorbid disorders using a published tool [2,7,8], in order to produce a comprehensive analysis of the biological and biochemical pathways as well as the evolutionary constraints on these gene sets. We deployed a bioinformatics strategy to robustly characterize disease genes, cluster these disorders to find those diseases most closely related to autism, and explore the intersection. We also performed a network analysis in order to target key functions and determine the most highly connected nodes in pathways unique to ASD. By using this prior knowledge of genes associated with ASD and its related comorbid disorders, we test here whether we can find novel aspects of autism gene candidates, analyze the properties of the gene set involved in ASD only, its singular evolutionary constraints and its relevance to the molecular pathologies of autism.

## Materials and Methods

### Research of comorbid disorders associated with Autism Spectrum Disorder

We used the results of three research studies that investigate comorbid disorders that occur at a significantly higher frequency in individuals diagnosed with ASD than in an age-matched control population, as based on a population derived sample [1,3,9], electronic records [2–6], as well as a review paper [3,10]. We extracted all the ICD-9 codes of disorders comorbid to autism in these papers. When ICD-9 code lists were not directly available, we mapped the comorbid disorder terms mentioned in the paper to the closest ICD-9 code. For example, “depressive disorder” was matched to ICD-9 code 296.3 “major depressive disorder recurrent episode.” ICD-9 code 300.22 “agoraphobia without mention of panic attacks” indicated in Kohane et al. [3–6] corresponds to the MeSH Term “Agoraphobia”.

### Extraction of disease-associated genes from the literature

To conduct PubMed queries to retrieve disorder-related genes, we used two robust disease-gene text mining tools, Phenopedia [1,4,8] and Genehawk [2,7,8]. Phenopedia is a web-based application that utilizes a database that is continually updated from PudMed to facilitate the exploration of the literature on human genetic associations, and provides summarized human genetic association information regarding either genes or diseases. The complete method has been described by Yu *et al*., [8]. Genehawk is a rule-based text-analytics algorithm with keyword matching that can extract target disorders and significant gene-disorder results described by the article, as well as the type of study itself. The complete method is available in Jung *et al*., [7]. Briefly, the first step consists of retrieving articles related to each target disorder by building a comprehensive PubMed query to retrieve disorder-specific research or review articles. Then, the identified gene symbols and their names in the literature are extracted from the text and mapped to yield unique identifiers. The last step assesses the significance of the articles and genes by ranking articles and terms based on the strength of publication and the structure of the article. A minimum Genehawk score of 1.0 was used to match potential gene candidates for autism. Genehawk was updated in July 2014 for this analysis. Since both Genehawk and Phenopedia utilize MeSH (Medical Subject Headings form from U.S National Library of Medicine) terms for their automated searching of PubMed, we matched each ICD-9 code in our comorbid disorder list to MeSH terms (S1 Table). For example, ICD-9 code 300.22 “Agoraphobia without mention of panic attacks” indicated in Kohane et al. [3] corresponds to the Mesh Term “Agoraphobia”. Finally we completed the list of genes involved in ASD by adding the genes from SFARI genes [11], as well as the gene sets from Iossifovet al., [4] and De Rubeis et al. [12].

### Statistical analysis and data visualization

**Fig 1:**
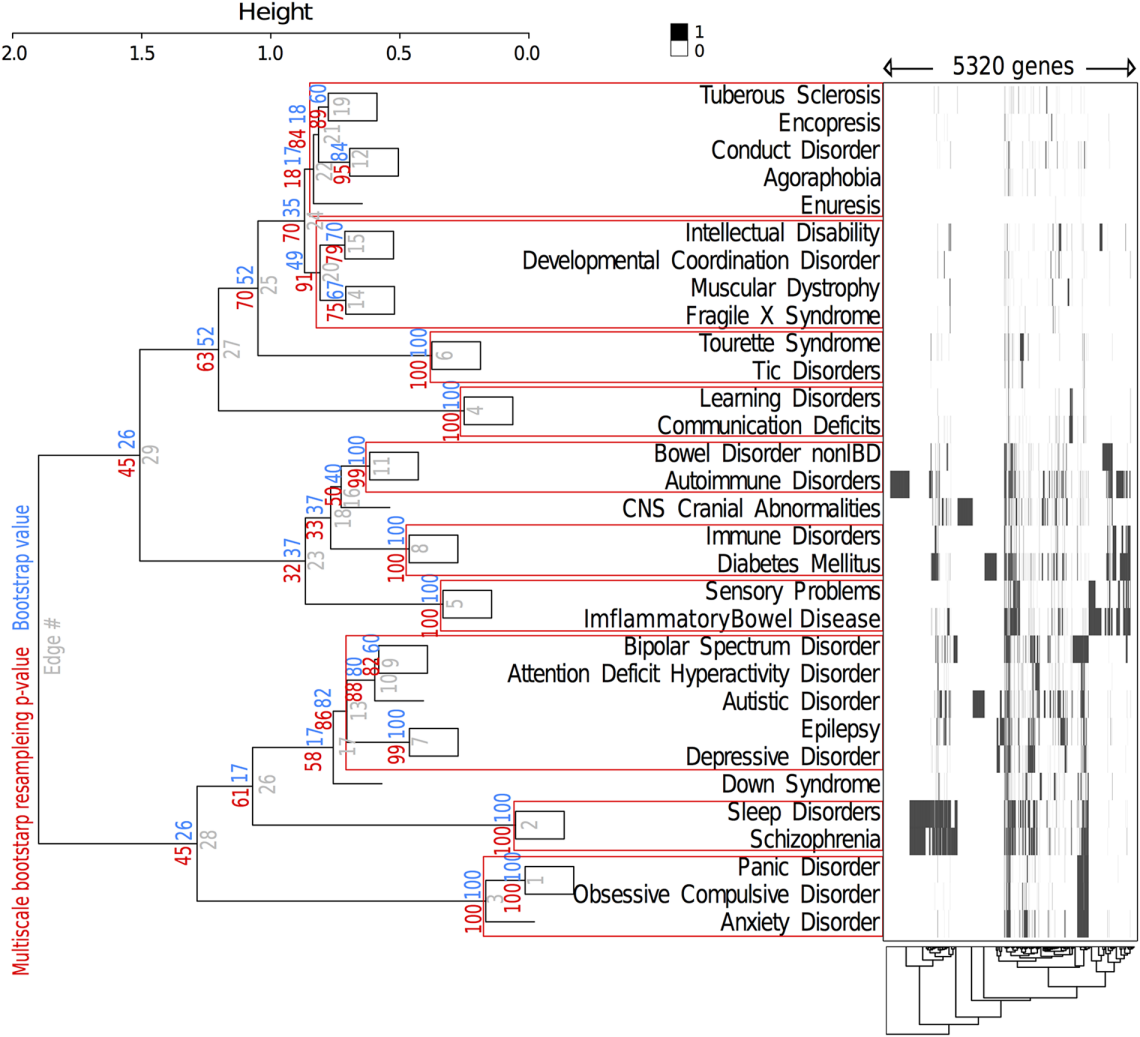
Hierarchical cluster analysis using the presence/absence matrix of genes associated with Autism Spectrum Disorders (ASD) and disorders comorbid to autism (complete list provided in S5 Table). Values at branches are *p*-values (in red) and bootstrap probabilities (blue) in percentage, and the clusters framed in red are supported by a *p*-value of 0.15. This approach revealed several clusters of disorders based on the genes they share with each other, including one cluster with ASD and five other disorders.

We performed all statistical analyses with R Studio Integrated development environment for R v0.97.336 (open source software, Boston, MA., http://www.rstudio.org/). Clustering analysis between disorders was performed on a presence-absence matrix of all the genes involved in all the comorbid disorders by calculating distances using a binary method and generating a hierarchical clustering using Ward D2 method (library pheatmap). To consolidate the clusters, we calculated bootstrap values using 1000 iterations. We also computed *p*-values with multi-scale bootstrap on 1000 resamplings using the package pvclust (Fig 1), which provides a better approximation of an unbiased *p*-value than bootstrap values calculated using normal bootstrap resampling [13,14]. To define our clusters we used a significance level of 0.15, meaning that the null hypothesis, i.e. "the cluster does not exist", was rejected, supporting that these clusters did not exist due to sampling error. The standard errors of each *p*-value were plotted in S1 Fig. The plots indicated that the standard errors were low and within an acceptable range, i.e 0.02 for the cluster threshold we used in our study (>0.85). Finally the fitting curves of each cluster are plotted in S2 Fig.

### R packages

We used the following R packages: Pheatmap, ggplot2.

### Functional characterization of genes related to ASD

In order to characterize the biological functions of the gene sets associated with the diseases previously defined in this study, we utilized the KEGG database [15]. We mapped the symbol IDs to KEGG Orthologs (KOs) using the KEGG API (http://www.kegg.jp/kegg/rest/) (S2 Table). We extracted metabolic KEGG pathways using the KOs identified, (S3 Table), and estimated the pathways with the highest ASD gene coverage by comparing the number of KOs we found to be correlated with ASD and to the total number in the pathway.

### Network analysis

**Fig 2:**
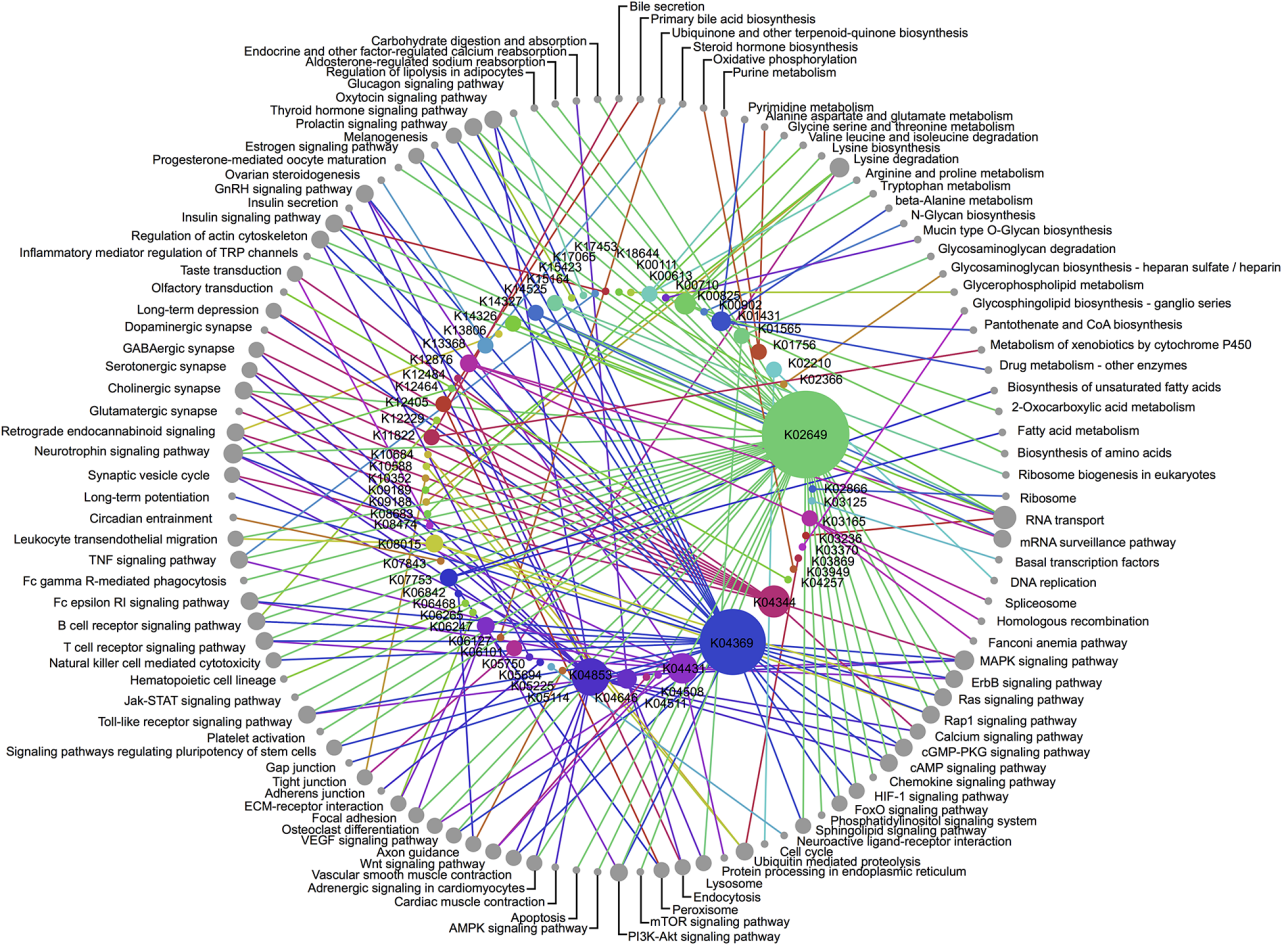
Network analysis of the KEGG Orthologs and the pathways with which they are associated in the KEGG database. Each node in the inner circle represents a KEGG Ortholog and each node in the outer circle indicates the pathway in which each KO is involved. The size of each node is proportional to its connectivity.

We performed a network analysis using GEPHI and the dual-circle layout using the circular layout plug-in (Fig 2).

### Genomic Evolutionary Rate Profiling (GERP)

For our GERP analysis, we downloaded the orthologous alignment scores used by Cooper *et al*., [16] (available at http://mendel.stanford.edu/SidowLab/downloads/gerp/). We used the gene file indicating each coordinate made available by NCBI to map, in R, our gene lists to the corresponding regions showing significant evolutionary constraint (threshold at *p*-value of 0.05).

### dN/dS value

Human and other primate sequences corresponding to our ASD and comorbid disorder genes were used to calculate the dN/dS ratio of non-synonymous (dN) to synonymous (dS) nucleotide substitutions of the orthologous coding sequences (CDS) present in either the gene sets associated with ASD only or with comorbid disorders, in order to assess the evolutionary characteristics of each of these genes in the primate lineage. The primate dN/dS ratios were measured with codeml from the PAML software by using best reciprocal coding sequence alignments of 9 primates including human, chimpanzee, gorilla, orangutan, gibbon, macaque, baboon, marmoset and bushbaby. To obtain the alignments of best reciprocal CDS we used a combination of Blat [17] and PRANK [18] [19]. Ensembl v69 [20] human CDS were blatted on the non human primate genomes of chimpanzee panTro4, gorilla gorGor3, orangutan ponAbe2, gibbon nomLeu3, macaque rheMac3, baboon papAnu2, marmoset calJac3, and bushbaby otoGar3. Best reciprocal hits were then identified by blatting the best matches on the human genome hg19. Best reciprocal CDS were then aligned using the PRANK codon evolution model. All the ratios are provided in S4 Table.

### pN/pS value

We extracted the pN and pS values for 1000 genome project (http://www.1000genomes.org/data). pN indicates the degree to which two sequences differ at non-synonymous sites within a population (here the African human genome from 100genomes project), i.e. the number of non-synonymous polymorphisms per gene. pS is a measure of the degree to which two homologous sequences differ with respect to silent nucleotide substitutions (substitutions that do not cause an amino-acid substitution). To calculate the pN/pS ratio, we divided the mean of all the pN values of the gene sets considered by the mean of all the pS values of the gene sets being compared (S4 Table). We did a random sampling of the gene sets presenting the lowest number of genes to generate a distribution and compared this to the ratio of the smallest group. For example, to compare 262 genes involved in ASD only with the genes involved in ASD and another disorder, we randomly sampled 262 genes in the larger comparison dataset, and performed this sampling 1000 times. We then used the value mean pN/mean pS of the smallest gene set to determine the threshold, and generated the *p*-value by taking the sum of the values above the threshold divided by the sum of all the values observed in the distribution.

### KEGG analysis

The KEGG database was interrogated using the available KEGG API [15].

### Eutils and NCBI APIs

To retrieve NCBI related information for each gene we used the NCBI API eutils. [21]

## Results

### Overview of the genes involved in ASD and comorbid disorders

A literature review allowed us to find 125 ICD-9 codes comorbid to Autism Spectrum Disorder (ASD), which we consolidated into 31 disorders (S1 Table). Using automatic literature searches—Genehawk [7] and Phenopedia [8]—we extracted as few as three genes (for Enuresis) and as many as 1583 genes for Schizophrenia (S5 Table), depending on the literature available on each disorder. We found 1031 genes involved in ASD, among which 262 seem to be involved in ASD only and 779 in other disorders as well. Both sets of genes were present in all the 22 autosomes, and did not show any significant different in length between the two sets. In order to visualize these gene sets, we performed clustering on a binary matrix of gene presence/absence (Fig 1) [22] [23]. This approach allowed us to define cluster of disorders based on their shared and associated genes. We extracted a statistically supported cluster showing that several genes are shared among ASD, Depressive Disorder, Bipolar Spectrum Disorder, Attention Deficit Hyperactivity Disorder, and Epilepsy.

### Pathway analysis associated with ASD

**Fig 3:**
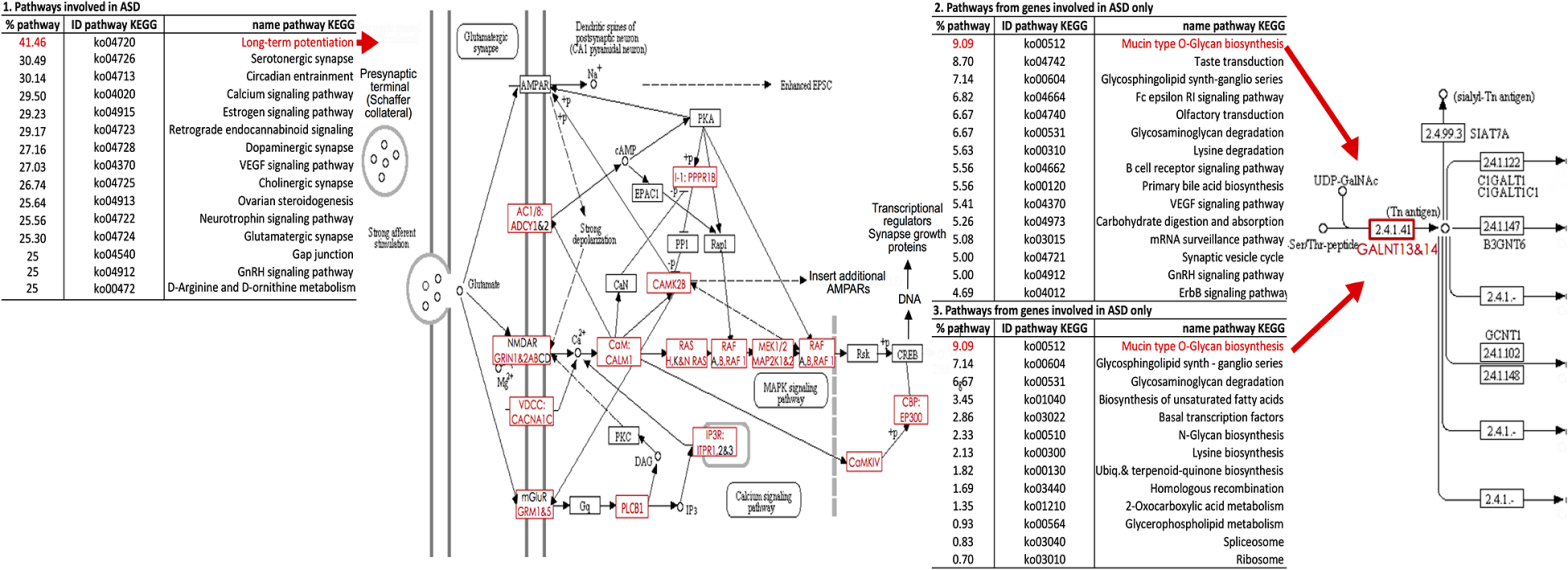
Analysis using KEGG database of the pathways associated with: 1) all the genes involved in ASD, 2) the genes involved in ASD only and 3) the pathways associated with ASD and no other comorbid disorders. The percent indicates the number of KEGG Orthologs detected with this analysis in comparison to the total number of KEGG Orthologs of this pathway, i.e. the percentage of the pathway covered in this analysis. For 1) and 2) we show only the top 15 pathways.

The identification of genes uniquely related to ASD allowed us to distinguish metabolic and regulatory pathways putatively specific to this condition. To do so, we used the 1031 symbol gene IDs retrieved from our literature search; 737 mapped to KEGG Orthologs (KO) involved in 194 KEGG pathways (Fig 3, S2 and S5 Tables). Fig 3 shows that genes known to be involved in ASD as well as in other conditions cover roughly 40% of pathways like long-term potentiation, while the genes involved in ASD only cover almost 10% of Mucin Type O-Glycan biosynthesis. Several of these pathways are involved in synaptic functions, more specifically: Serotonergic, Dopaminergic, Cholinergic, Glutamatergic and endocannabinoid signaling (synapse retrograde messengers). We also observed pathways directly involved in neural function, namely the neurotrophins signaling pathway (a family of neurotrophic factors involved in differentiation and survival of neural cells) and the hippocampal long-term potentiation (LTP) pathway (41.6% of KOs associated with ASD), which constitutes the molecular basis for learning and memory in the hippocampus. This latter pathway has been associated with ASD in numerous studies, e.g., abnormalities in the circadian cycle have appeared in a mouse model of autism as well as in the calcium signaling pathways [24]. We also observed three hormonal pathways associated with autism: the estrogen signaling pathway, for which the beta receptors were found to be disturbed in subjects with autism [25], the ovarian steroidogenesis pathway [26], and Gonadotropin-releasing hormone (GnRH) secretion pathway, which acts upon its receptor to release the gonadotropins and by cascade reactions acts on mitogen-activated protein kinases (MAPKs) pathways [27]. Anecdotally, these pathways could relate to congenital malformations of the reproductive system in males [28]. In addition, we found two pathways that mediate cell responses: the VEGF signaling pathway, which is a major player in vascular permeability as it moderates endothelial cell responses and their proliferation [29], and the gap junction pathway, which contains intercellular channels that allow direct communication between the cytosolic compartments of adjacent cells [30]. Finally, we identified a pathway related to amino-acid production: D-Arginine and D-ornithine metabolism that is also uniquely associated with ASD based on our gene sets; variants associated with ASD in the ornithine transport system.

### Pathway analysis associated with ASD only

Among these pathways, 113 were extracted from the 262 genes involved in ASD only, and we observed thirteen pathways not involved in any other comorbid disorders and therefore unique to ASD. While we did not detect an entire pathway associated exclusively with ASD, Mucin type O-Glycan biosynthesis shows several genes associated with ASD. Mucins are glycoproteins that are ubiquitous in the human body. They are present in mucous secretions on cell surfaces and in fluids and interact with α-Neurexins, a type of presynaptic cell surface molecule essential for neurotransmission and linked to neuro-developmental disorders such as autism and schizophrenia [31]. Sphingolipid biosynthesis is another interesting pathway that appeared to be enriched among the genes unique to autism and not found in disorders comorbid with autism, potentially due to its role in maintaining membrane fluidity as well as the integrity of lipid rafts and Glycerophospholipid metabolism [32], which is the case as well for the biosynthesis of unsaturated fatty acids pathway. The Glycosaminoglycan pathway has already been suggested as a biomarker for autism as it may potentially be involved in the etiology of the disorder with an aberrant extracellular matrix glycosaminoglycan function localized to the subventricular zone of the lateral in subjects with ASD [33]. The N-Glycan is synthesized by processing a protein’s oligosaccharide moiety (N-glycan) and serves several functions for proper central nervous system development and function. Previous experimental and clinical studies have shown the importance of proper glycoprotein sialylation in synaptic function in autism spectrum disorders (ASD) [34]. We also noticed generic functions such as basal transcription factors, homologous recombination, ribosome and spliceosome, which have all been associated with autism [35]. Several amino-acids, especially lysine, have been associated with ASD when examining the plasma level [36], which is also the case for the co-enzyme Q (from the ubiquinone biosynthesis pathway).

### Network analysis of genes involved in ASD only

Interconnected genes unique to ASD are of high interest, as any variation in their coding sequence will impact the function of pathways detected with this analysis, including several of the pathways uniquely connected to ASD. To determine whether genes unique to ASD appeared to be involved in specific pathways, we performed a networks analysis (Fig 2). K02649 showed the highest connectivity and is part of the regulatory subunit of a phosphoinositide-3-kinase. Activated by many types of cellular stimuli, this KO regulates fundamental cellular functions such as transcription, translation, proliferation, growth, and survival. In addition, this KO is a key component of the regulatory system as it is involved in 71 other pathways. The second most connected KO (K04369) was the mitogen-activated protein kinase. The MAPK signal transduction pathways are among the most widespread mechanisms of cellular regulation and are known to be associated with ASD [27]. To a lesser extent, three more KOs appeared to be involved in several pathways and associated with ASD only. K04431 (present in 12 pathways) is a mitogen-activated protein kinase as well; the other two proteins are from voltage-dependent calcium channels: K04344 (implicated in 14 pathways) K04853 (present in 20 pathways). All three of these KOs are part of the MAPK signaling pathway.

### Genomic Evolutionary Rate Profiling

**Fig 4:**
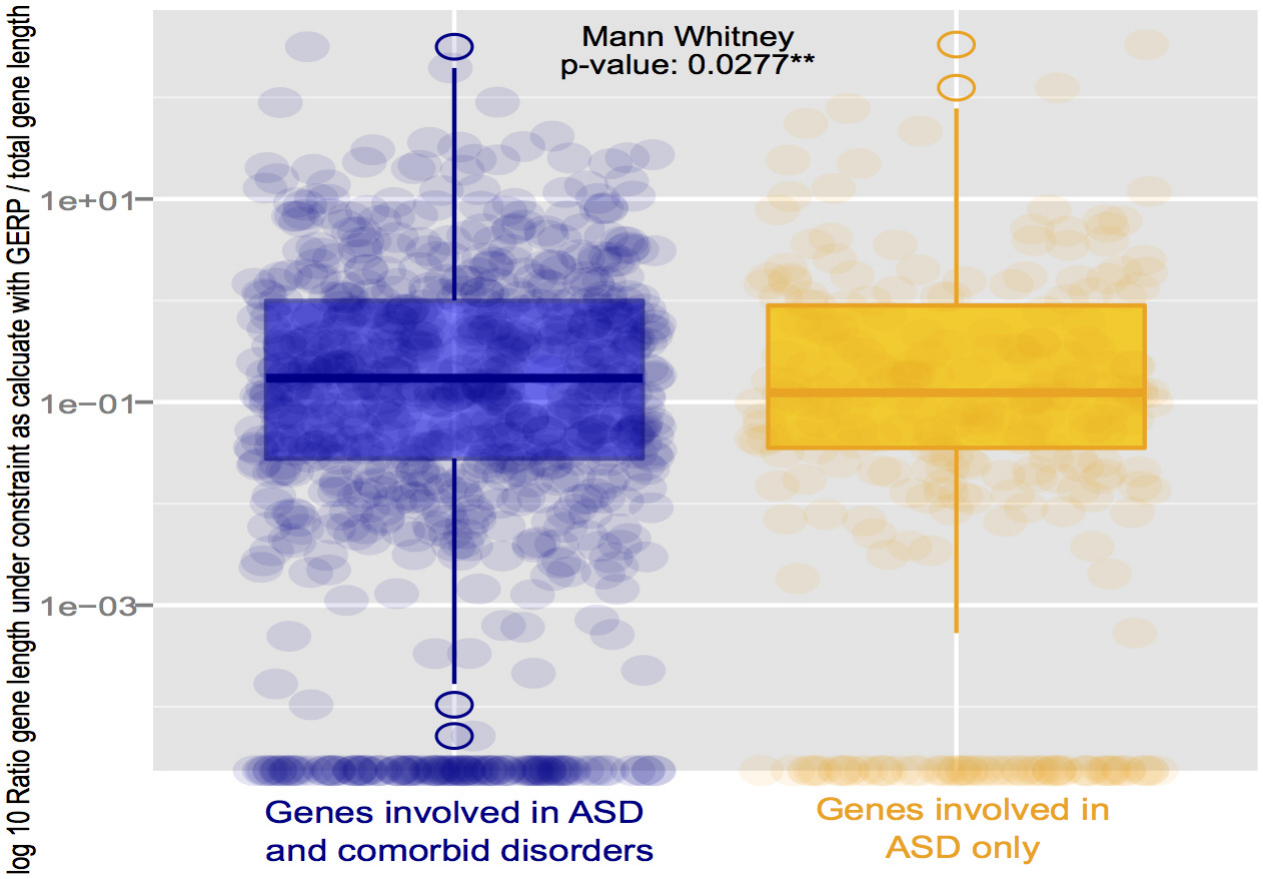
Boxplot showing the length of nucleotide sequence under constraint (using Genomic Evolutionary Rate Profiling) normalized by the total length of each set. A Mann Whitney test was used to test if the genes involved in ASD alone have undergone more purifying selection than the gene involved in comorbid disorders.

In order to determine whether the regions involved in ASD only have been subject to purifying selection and are enriched for functional elements, we compared orthologous genomic DNA sequences by using Genomic Evolutionary Rate Profiling (GERP) [16]. This method compares orthologous genomic DNA sequences by aligning sequences from 29 mammalian species to characterize regions that have been subject to purifying selection and identify constrained elements. A Mann Whitney test was used on a length of nucleotide under constraint normalized by the total length of each set. The results (Fig 4) indicate that the genes involved in several comorbid disorders seem to have undergone more purifying selection than the genes unique to ASD, as shown by the higher GERP score (significant difference with Mann-Whitney test p-value: 0.0277).

### dN/dS ratio

**Fig 5:**
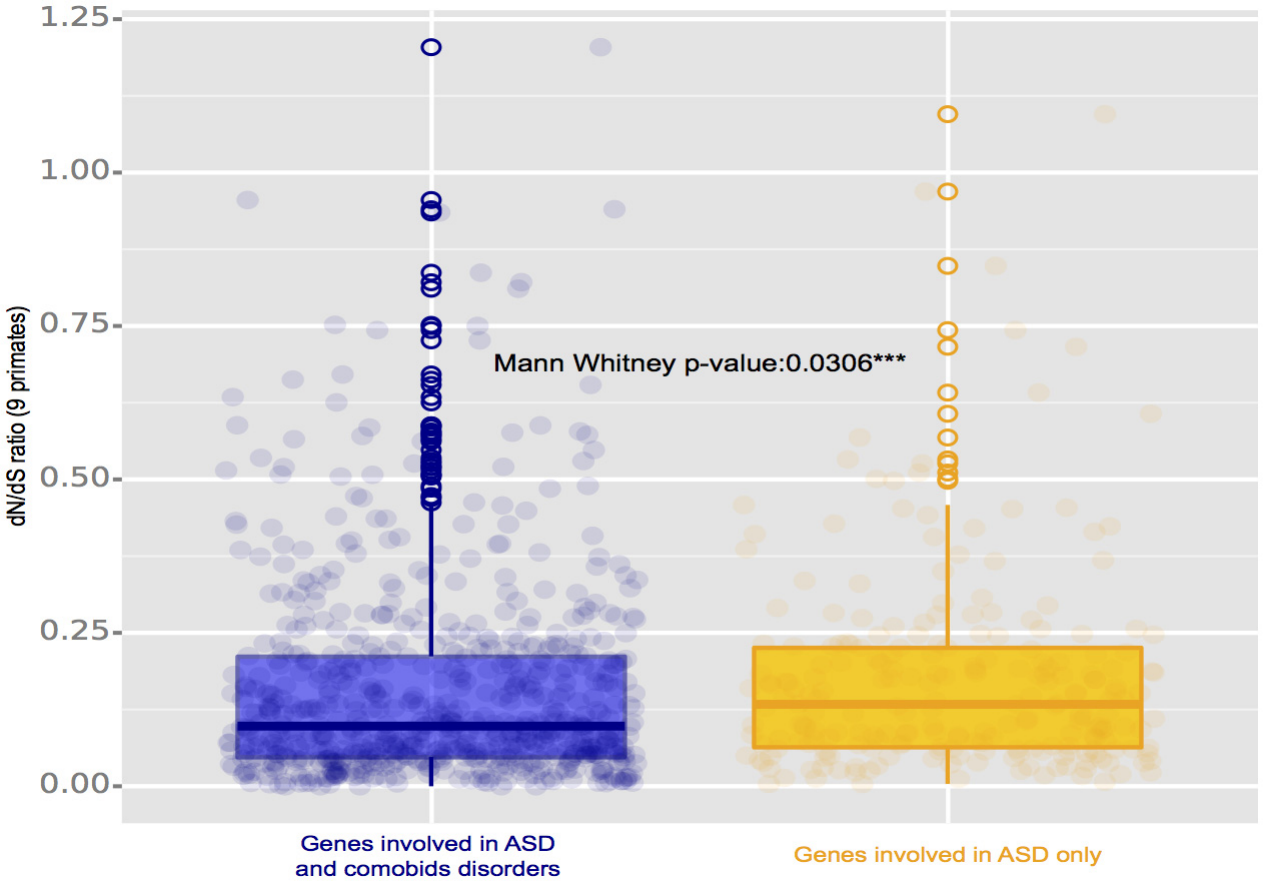
Boxplot of the dN/dS ratio calculated from the alignment of 9 primates including human, chimpanzee, gorilla, orangutan, gibbon, macaque, baboon, marmoset and bushbaby. The results of the Mann Whitney test showed that genes involved in several comorbid disorders have undergone more purifying selection than the genes uniquely associated with ASD. This observation is consistent with the GERP results in Fig 4.

Human and other primate sequences corresponding to these genes were used to calculate the dN/dS ratio of the orthologous coding sequences present in either the gene sets associated with ASD only or with comorbid disorders. Comparing the dN/dS ratio of genes involved in ASD with the ones involved in several disorders shows a significant p-value of 0.0306 (Fig 5); this ratio was significantly higher for ASD-only genes.

### pN/pS ratio

**Fig 6:**
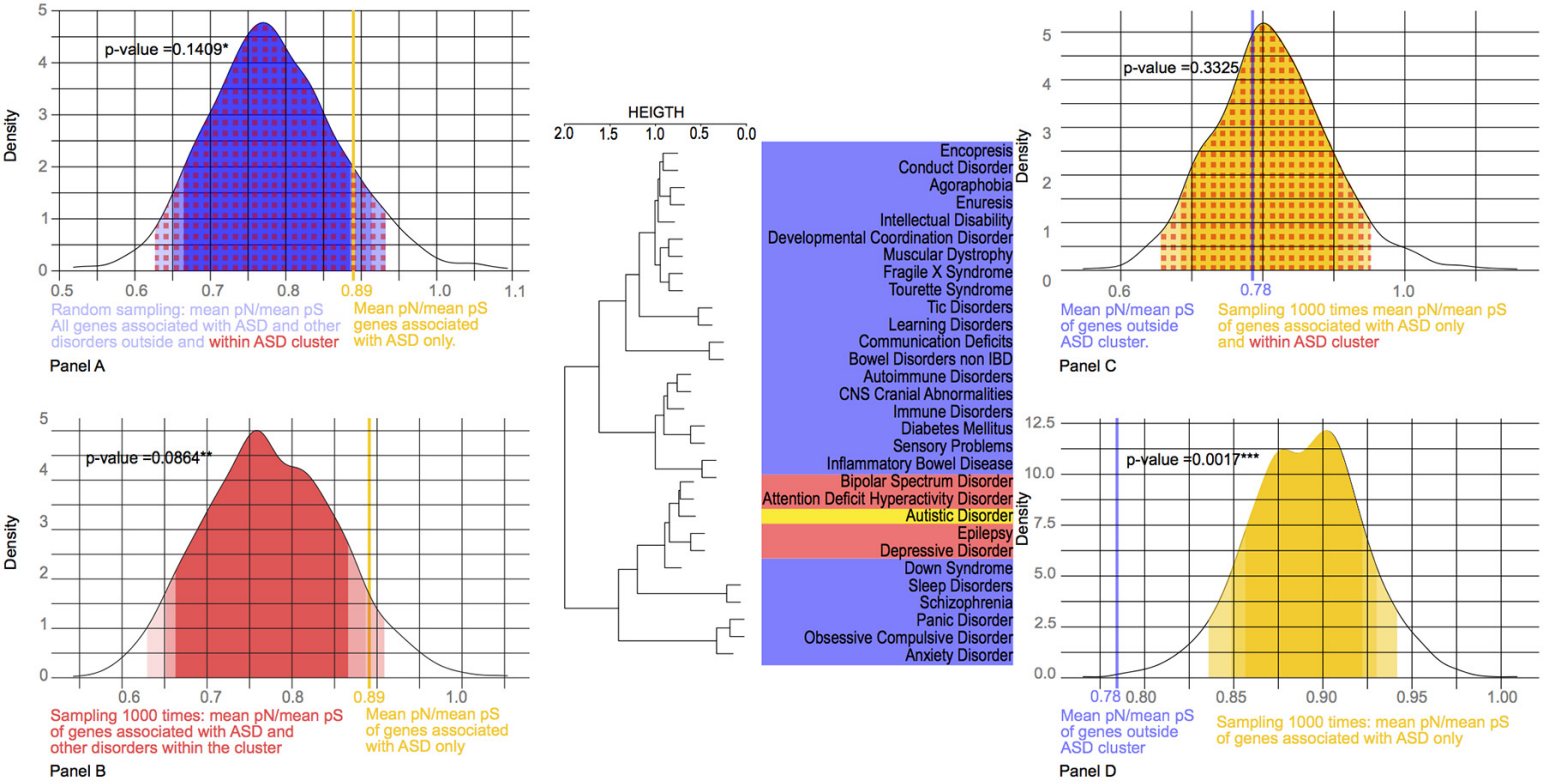
Distribution of 1000 resamplings of the mean pN/ mean pS ratio of the genes involved in ASD (yellow), comorbid disorders within the ASD cluster (red) and other comorbid disorders (blue). The different panels show comparisons between: genes associated with ASD only (yellow) versus all other comorbid disorders (red+blue) (Panel A), genes associated with ASD only (yellow) versus comorbid disorders within the ASD cluster (red) (Panel B), genes associated with ASD and comorbid within the cluster (yellow + red) versus the genes outside the cluster (blue) (Panel C), genes associated with ASD only (yellow) versus genes associated with ASD outside the ASD cluster (blue) (Panel D).

While the dS/dN ratio reflects the evolutionary history among mammals, it does not inform us about the more recent evolution among humans. To analyze recent human-specific constraints, we used the pN/pS ratio. We compared this ratio for genes uniquely associated with autism to those genes shared between ASD and other disorders (Fig 6, panel A) and showed that both gene sets seem to be under strong purifying selection with pN/pS < 1. However, the gene set involved in several disorders shows higher constraints than the genes involved in ASD only, with a mean of 0.89 for the (mean PS)/(mean pN ratio) for the genes associated with ASD only. Fig 6 (panel B) compares the pN/pS ratio of the genes associated with ASD only and the ones within the ASD cluster, and shows the same trend with a significantly elevated ratio for the genes associated with ASD. We performed the same analysis by comparing the genes associated with ASD only and the ones outside the ASD cluster (Fig 6, panel D), and again the ratio was significantly different, showing than the genes shared across the ASD and comorbid disorders are under higher evolutionary pressure than the genes unique to ASD. Finally, to test whether the pN/pS signal was observed in the whole cluster, we compared the pN/pS ratio of the whole cluster against that of the genes outside the cluster (Fig 6, panel C).

### Cognitive disorders analysis

We extended the analysis to any genes involved in comorbid disorders associated with cognitive function, i.e.: Conduct Disorder, Agoraphobia, Developmental Coordination Disorder, Intellectual Disability, Tourette Syndrome, Tic Disorder, Learning disorder, Communication Deficit, Attention Deficit Hyperactivity Disorder, Epilepsy, Depressive Disorder, Down Syndrome, Schizophrenia, Panic Disorder, Obsessive Compulsive Disorder, and Anxiety Disorder. We explored the pN/pS ratio comparing this set of cognitive disorders with the set of genes involved in ASD only, and obtained a p-value of 0.1218. The same ratio of all cognitive disorders to non-cognitive disorders (i.e. Muscular Dystrophy, Bowel Disorder non IBD, Autoimmune Disorders, CNS Cranial Abnormalities, immune Disorders, Diabetes Mellitus, Inflammatory Bowel Disease and Sleep Disorders) did not show any differences (p-value = 0.3911). We performed the same analysis for dN/dS ratio and did not find any significant differences.

## Discussion

### Genes associated with ASD and comorbid disorders

Our search for genes using a previously published literature mining tool [7] found 1031 genes associated with ASD and at least one other related disorder, and a core set of 262 genes unique to ASD. Twenty-one of the genes unique to autism overlapped with the highest priority candidates reported by DeRubeis et al., [12] (N = 22) and Iossifov et al. [4] (N = 27), which focused on analysis of high coverage exome data from 3,871 and 2,517 autism families respectively.

### Functional analysis of genes associated with ASD

Of the pathways previously linked to ASD, we identified 13 pathways uniquely involved in ASD. While the pathways related to neural functions seemed to be associated with disorders comorbid to ASD, lipid biosynthesis and regulation pathways were associated with autism only. We also observed that basal cellular functions (ribosome, spliceosome, endothelial cells migration and signalization) appeared to be more specific to ASD. The coverage of the pathway involved in ASD was less extensive than that of pathways involved in several disorders, but the network analysis reveals two KEGG Orthologs with ubiquitous functions that could constitute ASD biomarkers: K04369 (genes MAP2K2, and MEK2) and K02649 (PIK3R gene). The MAPK pathway was previously reported to be involved in ASD and other disorders; here we have shown that it evidence for its unique association to autism, in comparison to with disorders comorbid with autism.

Evolutionary history of genes involved in ASD: We generated a set of genes and pathways implicated in only autism, all involved in basal cellular functions. This was in contrast to gene sets shared by ASD and comorbid disorders, which we found to be associated with neurological function. This suggested that the pathways specifically underlying autism were functionally distinct, and may have evolved (or be evolving) under different evolutionary constraints than genes shared between autism and comorbid conditions. To determine if these two gene sets showed discrepant evolutionary signatures, we looked for potential purifying or positive selection events during both mammalian evolution and more recent human history.

### Long-term evolutionary constraints

To characterize the evolution history of the genes involved in ASD, more specifically at the levels of evolutionary constraints, we first compared evolutionary constraints within mammalian phylogenies using GERP (Fig 4). We also determined dN/dS ratio within nine primates to compare orthologous genomic DNA sequences in order to determine whether the regions involved in ASD only have been subject to purifying or adaptive selection (Fig 5). The dN/dS ratio and GERP findings agreed: the genes involved in comorbid disorders had a lower dN/dS ratio indicating that they have undergone more purifying selection than genes unique to ASD. Other studies have observed higher evolutionary rates in ASD associated genes. For example, a ~35 Mb region on the chromosome at the loci 1q41-q42.2 which has been linked to autism [37] and covers five SNPs significantly associated with autism in the MARK1 gene which showed an elevated dN/dS ratio indicative of adaptive evolution [38].

### Recent evolutionary constraints

The pN/pS ratio was significantly different between the gene set within the cluster containing ASD and the set outside the cluster (Fig 6). Taking into account that some non-synonymous mutations are deleterious and the remainder neutral within the human African population, a pN/pS ratio less than 1 indicates strong purifying selection. Some amino-acid substitutions may have been caused by positive selection, but not enough to overcome the effects of purifying selection. Our data demonstrated that the genes involved in ASD only showed weaker negative evolutionary constraints than the genes involved in comorbid disorders. We also observed this result when comparing the ASD with the comorbid disorders present in its cluster (Fig 1), which contained other disorders related to neural and behavioral dysfunction: significant differences are still observed within this cluster. Moreover, we decided to explore if this signature was consistent when comparing ASD genes with genes involved in cognitive comorbid disorders: we did find that ASD was under less evolutionary constraint than all other cognitive disorders (Fig 6).

The literature has shown that coding sequences expressed in the brain evolved at a slower rate than in the rest of the genome [39]. This evolutionary measurement is consistent with the observation that ASD comorbid associated genes are under higher constraints than genes related to ASD only, and are implicated in pathways expressed in other locations than the brain. This finding can also be put into perspective by a previous paper by Keller & Miller [40], which evaluated 3 potential mutational models to explain the disconnect between the fitness cost and prevalence of mental conditions in humans: 1) ancestral neutrality (2) balancing selection and (3) the polygenic mutation-selection balance. They determined that a polygeneic mutation selection balance model best fit the data, which could be consistent with our finding that genes unique to ASD have reduced purifying selection in comparison to those genes shared between autism and autism-comorbid disorders. As such these autism unique genes may experience higher rates of mutations linked to cognitive changes observed in recent human evolution.

## Supporting Information

S1 Fig Standard error of each p-value calculated in Fig 1 multi-scale bootstrap. (PDF)

S2 Fig Fitting curve for the multiscale bootstrap performed Fig 1 for each cluster. (PDF)

S1 Table Correspondence table for ICD-9 codes, ICD-9 disorder, Phenopedia terms and MeSH terms. (XLSX)

S2 Table Correspondence between KEGG Orthologs, hsa (KEGG) and Symbol ID. (XLSX)

S3 Table Complete list of pathways described in Fig 3. (XLSX)

S4 Table pN value, pS value, and dN/dS ratio used in this study. (XLSX)

S5 Table Complete list of genes involved in each disorder analyzed. (XLSX)
