# Supplementary material for: Comorbid Analysis of Genes Associated with Autism Spectrum Disorders Reveals Differential Evolutionary Constraints
Source: PLoS One. 2016 Jul 14;11(7):e0157937. doi: 10.1371/journal.pone.0157937 (PMC4945013; doi:10.1371/journal.pone.0157937)
Supplement: S1 Fig — (PDF) [file pone.0157937.s001.pdf]

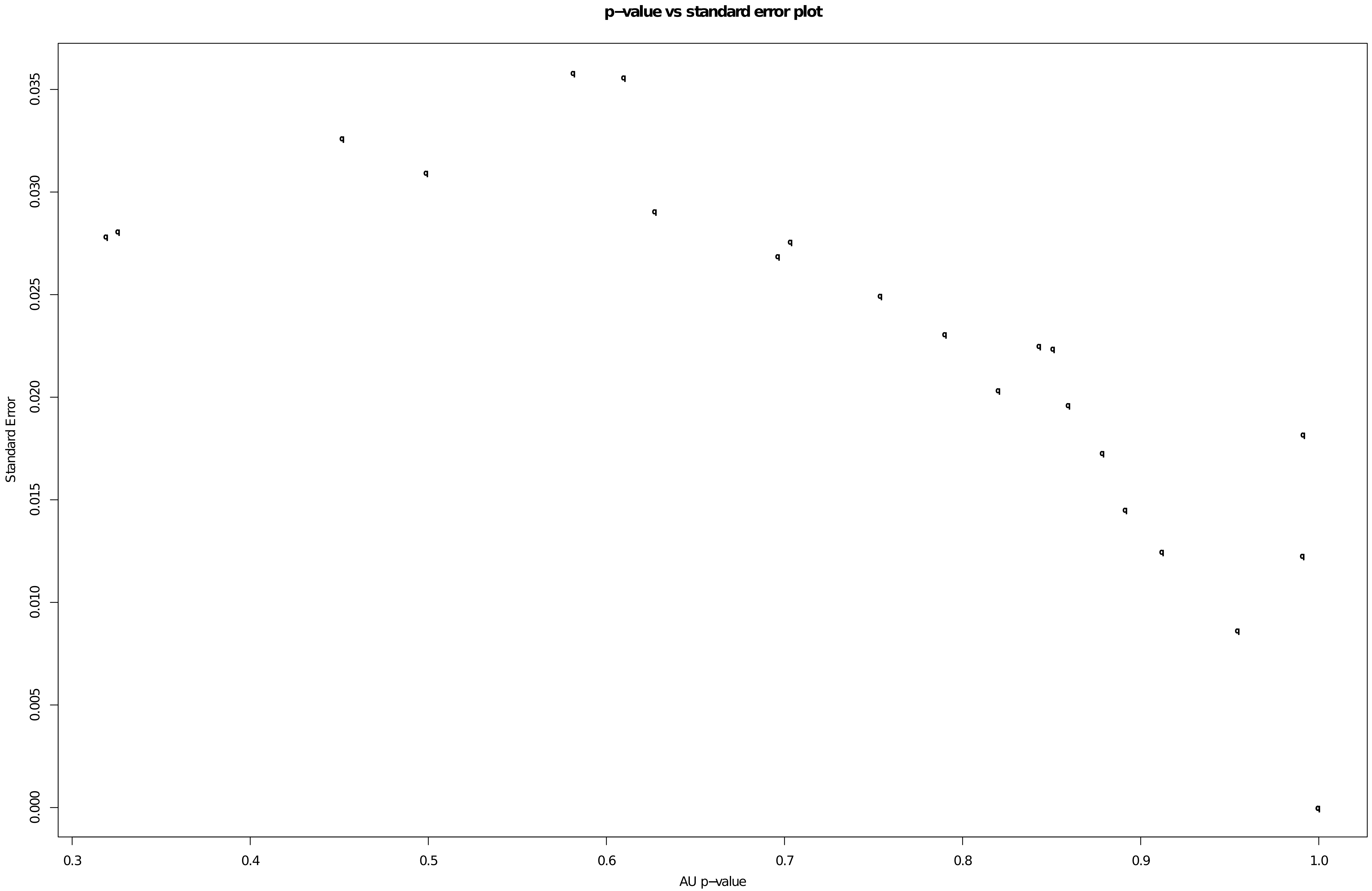

S1 Fig: Standard error of each p-value of multi-scale boostrap in Fig 1
