# Supplementary figures and images for: Comorbid Analysis of Genes Associated with Autism Spectrum Disorders Reveals Differential Evolutionary Constraints
Source: PLoS One. 2016 Jul 14;11(7):e0157937. doi: 10.1371/journal.pone.0157937 (PMC4945013; doi:10.1371/journal.pone.0157937)

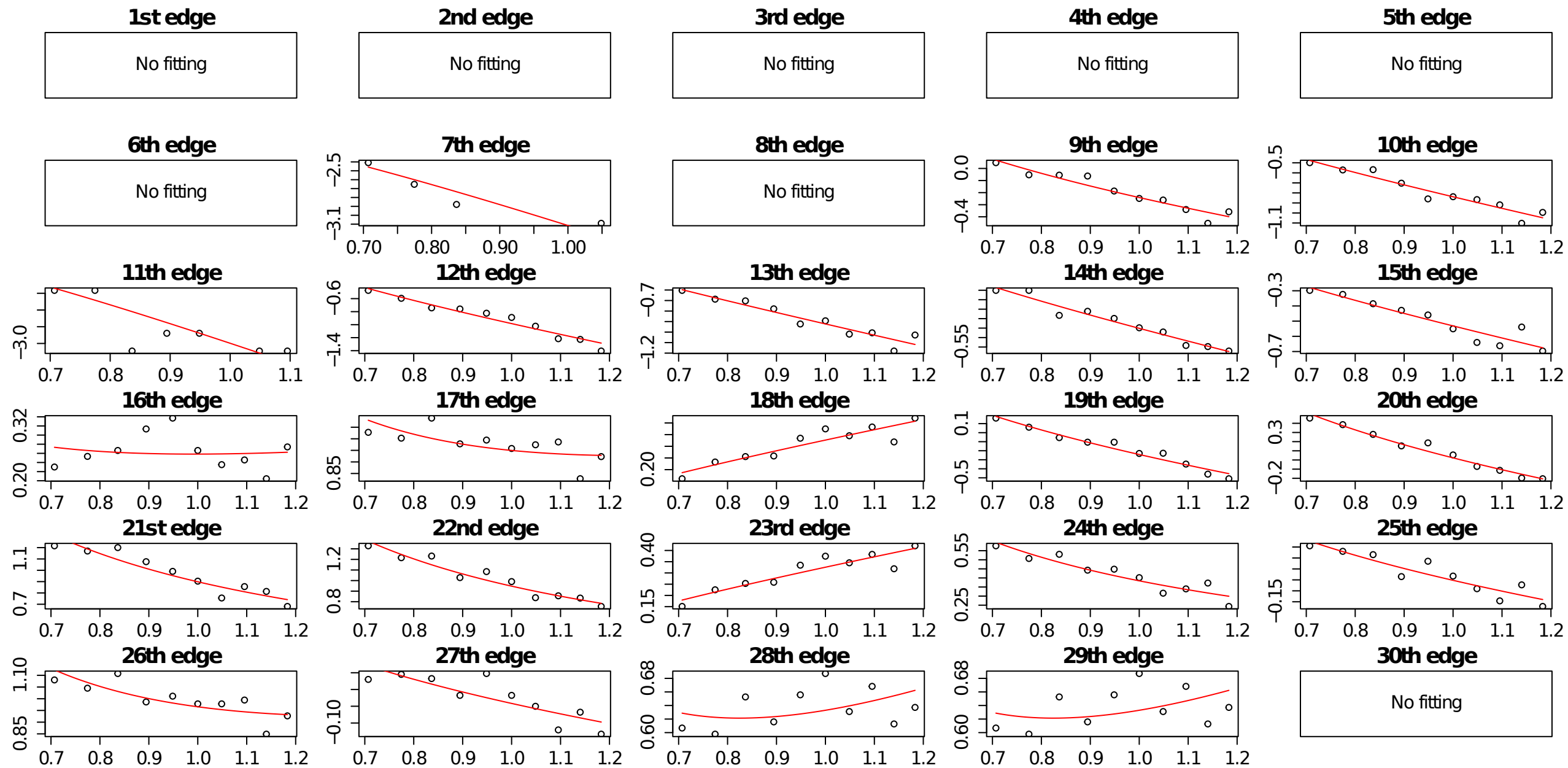

**S2 Fig:** Fitting curve for the multiscale bootstrap performed Figure 1 for each cluster

Supplement: S2 Fig — (PDF) [file pone.0157937.s002.pdf]
